# FEM-based time-reversal enhanced ultrasonic cleaning

**DOI:** 10.1016/j.ultsonch.2021.105798

**Published:** 2021-10-15

**Authors:** Joonas Mustonen, Oskari Tommiska, Axi Holmström, Timo Rauhala, Petro Moilanen, Maria Gritsevich, Ari Salmi, Edward Hæggström

**Affiliations:** a Electronics Research Laboratory, Department of Physics, University of Helsinki, P. O. Box 64, 00014 Helsinki, Finland; b Altum Technologies, Finland; c Finnish Geospatial Research Institute (FGI), Geodeetintie 2, 02430 Masala, Finland; d Institute of Physics and Technology, Ural Federal University, 620002 Ekaterinburg, Russia

## Abstract

Pipe fouling is a challenging problem in many industrial applications. Current cleaning techniques require halting the production during the cleaning phase and the existing methods are unable to do targeted cleaning, even though fouling is often localized to certain areas inside the pipeline. To address this issue, we use FEM-simulated, time-reversed signals to focus ultrasound power onto a pre-determined location: a fouled pipe residing inside a Plexiglas container. Ultrasound cleaning with similar acoustic power was compared to the time-reversal enhanced method in terms of cleaning efficiency. The cleaning efficiency was determined by measuring how much fouling, by mass, both protocols removed from the surface of a Plexiglas pipe, using similar input electric power and equal cleaning time. Our results indicate that the proposed time-reversal-based technique removes three times more fouling than the standard ultrasound cleaning without focusing. The study extends our previous paper on FEM-based time-reversal focusing [1].

## Highlights

• We introduce a time-reversal enhanced ultrasonic fouling removal method.
• The technique enables localized cleaning, using FEM-based time-reversed signals.
• Time-reversal enhanced technique removes fouling locally and efficiently.

## 1 Introduction

In many industrial applications, fouling accumulates with time inside equipment, such as pipelines, heat exchangers, and processing tanks. Some of these equipment feature complex internal structures. In most cases, fouling decreases the production efficiency and creates a need for maintenance and cleaning. The industry has developed techniques to address the problem, based on chemical and mechanical cleaning protocols [2]. Unfortunately, these methods require interrupting the production [2], which causes economic loss. Moreover, the employed chemicals often have harmful environmental impact [3].

In 2006, Nakagawa *et al.* introduced an ultrasound-based method for pipe cleaning [4]. An ultrasound-based approach avoids production stoppages and use of polluting/toxic chemicals [5]. Subsequently, companies have commercialized the technique [6]. Chen *et al.* investigated the ultrasound-mediated antifouling effect for heat exchangers experimentally, with promising results [7]. Lais *et al.* utilized finite-element modelling (FEM) to investigate how to optimize ultrasonic cleaning in pipe structures by determining cleaning patterns of propagating sound waves [8]. Habbibi *et al.* applied an acoustic antifouling method based on guided waves to reduce accumulation of biofouling on ship hulls in an environmentally friendly manner [9].

Ultrasound can produce a bubble implosion phenomenon called inertial cavitation. Relying on this phenomenon, fouling can be detached due to forces exerted by shock waves that are generated by collapsing bubbles. During the rarefaction phase of the applied ultrasound, the local peak-negative-pressure may decrease below the vapor pressure causing a phase transition from liquid to gas. This process of bubble forming, oscillation and possible subsequent collapse is defined as cavitation [10]. Furthermore, the applied ultrasound can force bubbles in the liquid to oscillate, if the frequency content of the present mechanical wave corresponds to the resonance frequency of the bubble, determined by the size of the bubble [11]. When the peak-negative-pressure is strong enough, the bubble radius can become more than twice as large as it is in the equilibrium condition without an applied external pressure wave [12]. In this case, the surface tension of the bubble can often no longer maintain the bubble integrity, which results in a bubble collapse due to the applied external pressure wave [13]. This is called inertial cavitation [14], [15]. The bubble implosion can create thousands of Kelvins of temperature and pressures in the range of gigapascals and, therefore, may damage solid surfaces immersed in the liquid [13]. Pečnik *et al.* investigated descaling mechanisms of cavitation-based cleaning and discovered two different erosion processes: eroding layer by layer (cohesion interaction) and cracking larger pieces of coated surface (adhesion interaction), depending on the composition and microstructure of the fouling [16]. They noted that the erosion strongly depends on the pressure amplitude of the applied ultrasound.

Ultrasound has been applied to remove fouling [4]. However, existing solutions are unable to focus the cleaning power, which is wasteful since the fouling is usually localized in industrial applications [17]. To apply cleaning power efficiently without causing unnecessary mechanical stress around the target (fouling area), spatial control of the cleaning power is required. Inability to focus the sound limits the cleaning efficiency even in simple structures like pipes.

**Fig. 1:**
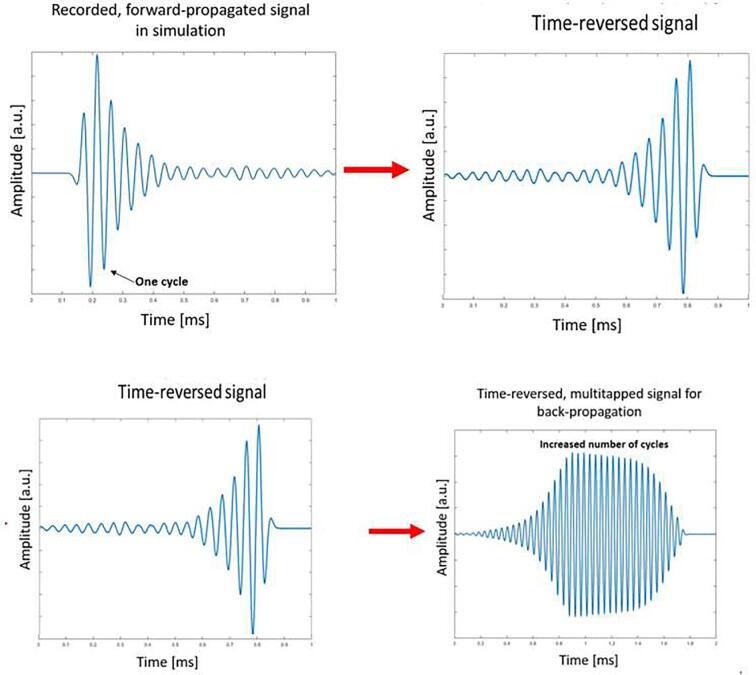
Time-reversing and multitapping the simulated signal. By flipping indices, A) signals become B) time-reversed and the result appears to be mirrored in time. By adding together several of these C) TR signals with a suitable phase shift, D) the multitapped signal is a superposition of several direct impulse actuations instead of one. This alters the shape of the signal by increasing the number of high-amplitude cycles.

In 1965, Clay and Parvulescu, introduced an ultrasonic transmitting method called *matched signals* to allow focusing with high signal-to-noise ratio in a medium with many scatterers, eg. bubbles, air pockets and other inhomogeneities, based on the reciprocity principle of the wave equation [18]. Later, Mathias Fink applied the same method by developing a time-reversal mirror (TRM) as an adaptive ultrasound focusing technique for maximizing the pressure at a pre-determined target location in an inhomogeneous medium [19]. Traditionally, in TRM, forward-propagation is conducted by direct impulse actuation (such as delta peak) at the target location and the propagated sound wave is recorded with surrounding transducers connected to e.g. an oscilloscope (Fig. 1). The signals are processed by time-reversing (flipping indices in time) and then transmitted ‘backwards’ from the transducers. Due to the reciprocity principle, the backwards propagated signals converges spatially and temporarily at the original target point, creating a focal spot. The method has been widely employed in medical applications, such as in lithotripsy and in brain hyperthermia, as well as for nondestructive testing [20]. The time-reversal technique has also been utilized in the field of electromagnetics, and it has enabled fault detection applications in communication and power networks [21].

In this work, we apply TRM as a focusing technique to improve the efficiency of ultrasonic cleaning. We demonstrate this ability by comparing cleaning results between a standard ultrasound cleaning method and the time-reversal enhanced method by measuring how much fouling mass both protocols remove from the surface of a Plexiglas pipe with equal input electric power and cleaning time. In TRM, first, the output of direct impulse actuation (such as a delta spike transmitted form the target location) is recorded. Then, the received signals are phase conjugated (i.e. ‘’flipped in time’). This creates the time-reversed signals [20]. In practice, this approach can be challenging if there is no physical access to the desired source location. We solve this issue by simulating the forward-propagation phase with COMSOL Multiphysics (5.4) [22]. In addition, generated time-reversed signals were post-processed by multitapping the signals, to maintain the pressure peak for a longer time than what is possible using merely one-cycle TR excitation.

## 2 Materials and methods

### 2.1 Fouling

The fouling used was a Ca-based paste, mimicking real industrial fouling. A mass of Ca(OH)_2_ powder and a volume of tap water were mixed in a 1 g : 1 ml ratio. Half of the powder was dissolved in water by continuously mixing the paste and after the mixture became homogeneous, the rest of the powder was added and mixed. The resulting paste was spread as a 1 mm thick layer on the outer surface of hollow Plexiglas pipes (300 mm long, outer diameter 20 mm, wall thickness 2 mm). The covered pipes were left to dry for 12 h before immersion and sonication.

### 2.2 Time-reversal and multitapping

The time-reversal process includes three steps: direct impulse actuation from a pre-determined focal point, recording the response with a phased transducer array, and retransmitting the time-reversed signals. According to the reciprocity principle, the back-propagation focuses the sound spatially onto the original source of the forward-propagation. In the time domain, the SNR of the focusing depends on the recording duration [23]. In this study, forward-propagated signals were generated by FEM-simulations (COMSOL Multiphysics 5.4). The FEM-model mimics the experimental setup, which features four narrowband transducers (f_c_ = 20 kHz, PZT-8, 100 W, Beijing Ultrasonics), coupled to a cylindrical Plexiglas container (diameter 300 mm and height 300 mm) in a symmetric configuration.

**Fig. 2:**
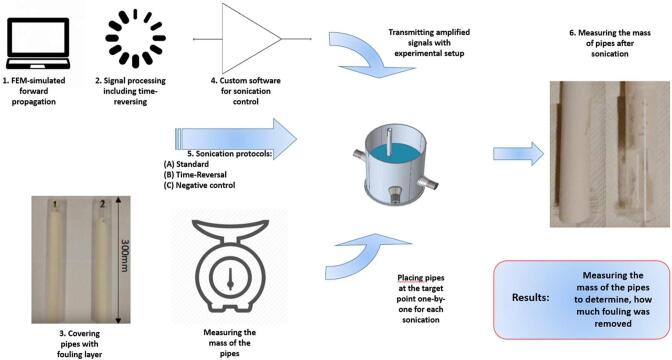
Diagram of cleaning process. (1) Forward-propagation was simulated with a FEM-model, (2) the obtained signals were processed and time-reversed, (3) pipes were covered with fouling and the mass of the pipes were measured, (4) cleaning process was controlled by the developed custom-made software, (5) pipes were immersed into water and sonicated one by one using one of three different protocols: standard, time-reversal enhanced and negative control, (6) the mass of the pipe was measured after the sonication to determine cleaning efficiency.

Simulated signals were processed by time-reversing and multitapping (Fig. 2). Time-reversing mirrors the signal in time by flipping indices (T: t → -t). The highest peak of the signal corresponds to the original direct impulse actuation in the forward-propagation and therefore occurs at the target point in the backward propagation, causing the cleaning effect. The time-reversed signals were multitapped (time-translated and copied): the obtained TR signal was duplicated. Then, the duplicate was phase-shifted by one cycle (period of the signal). Finally, these two signals were summed. As a result, the new signals created two or more delta peaks instead of one, due to the superposition principle. Repeating the method several times, the number of effective cleaning cycles increases. Here, the final processed signal contained 20 cycles.

The cleaning process was controlled by custom-made Python-software (version 3.5), which permits using coded signals and arbitrarily allows one to translate the cleaning target, alter the cleaning time, and pulse repetition frequency. The signals were transmitted from a sound card (ASUS Xonar U7, bandwidth 192 kHz), amplified with a high-power (6000 W) amplifier and conveyed through an impedance matching circuit to the transducers.

### 2.3 Cleaning protocols

**Fig. 3:**
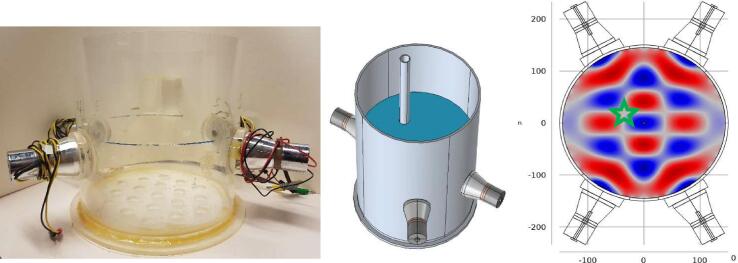
(Left) Plexiglass container. (Middle) Visualization of immersed pipe in the Plexiglas container (diameter 300 mm). In the real case, the pipes were covered by Ca(OH)_2_ fouling. The pipes were mounted on a separate Plexiglas plate placed on the top rim of the cylindrical vessel. (Right) FEM-simulation of standard protocol sonication in frequency domain. The green star indicates the target location, representing a node spot in the pressure field. (For interpretation of the references to colour in this figure legend, the reader is referred to the web version of this article.)

**Fig. 4:**
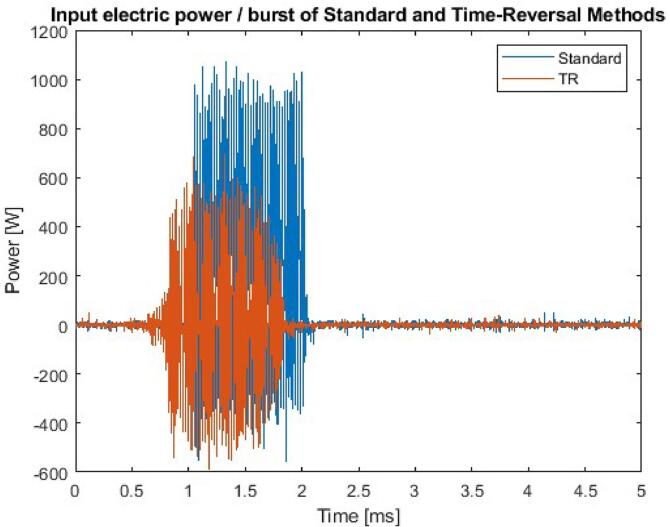
Example of input electric power/burst for both standard and time-reversal enhanced methods from one of the transducers. The total input electric energy was determined to be smaller with the time-reversal enhanced method, in favor of the standard method.

**Fig. 5:**
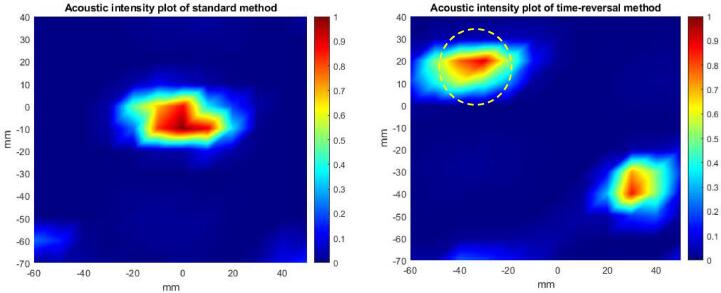
Experimentally determined acoustic fields of the standard protocol (left) and the time-reversal enhanced protocol (right), by mapping normalized intensities spatially at the time when the acoustic intensity peak occurs. The standard protocol has a focus at the center of the container (0 mm, 0 mm) and the time-reversal enhanced protocol has a focus at the target point (indicated with a yellow circle). The temporal peak-negative-pressure at the target point with the standard protocol was −550 kPa and −1030 kPa with the time-reversal enhanced protocol, implying the latter protocol should produce higher cleaning efficiency. (For interpretation of the references to colour in this figure legend, the reader is referred to the web version of this article.)

The pipe covered by fouling was immersed into the Plexiglas container holding room temperature water (Fig. 3). The location (the green star situated at the target location in the Fig. 3) was chosen based on frequency domain simulation analysis; simulations predicted that the long-term pressure should be relatively small (tens of Pascals) at the chosen location, representing a challenging industrial case. The pipe was sonicated for a pre-set time (60 s) with a 20-cycle sine pulse, here referred to as the ‘standard protocol’. The mass of the fouled pipe was measured before sonication and 12 h after the sonication to determine how much fouling was removed. The drying time of 12 h was selected to be sufficient to let the fouling dry completely. The procedure was repeated with three different samples. Then, the procedure was repeated with three other pipes by using the proposed time-reversal enhanced sonication technique, whose forward-propagation was produced at that location in the simulation model. The sonication time and electric input power were set to be equal in both experiments by using equally long bursts in both cases, using the same sonication time and by scaling the time-reversed input signals to have the same electric energy as the input signals in the standard protocol. In addition, as a negative control, three pipes were immersed in water for the same amount of time as the sonication protocol and the mass of the pipes were measured before and after immersion. No sonication was done in the control case Fig. 4, Fig. 5.

The aforementioned cleaning parameters (Table 1) were selected to make comparable cleaning experiments between TR-enhanced cleaning, standard cleaning, and negative control. For ideal comparison, the input energy / burst should be equal in the TR and in the standard protocol. However, as is seen in the fouling removal results, the difference between the control and standard protocols is rather small; by increasing the energy / burst levels in the standard protocol, we were able to discern a difference between the standard protocol and the control. Increasing the energy/burst of TR should yield improved cleaning. But as the significantly lower energy of the TR protocol already yielded higher cleaning efficiency, further increasing the energy/burst with TR was not deemed necessary.

**Table 1:** Cleaning parameters for each transducer. The input electric energy is higher with the standard protocol due to the shape and length differences between signals.

Cleaning parameters	
Center frequency	20 kHz
PRF	2 Hz
Cleaning time	60 s
Number of cycles	20
Input electric energy / burst (standard protocol)	250 mJ
Input electric energy / burst (TR protocol)	2 mJ

## 3 Results

Before conducting the actual cleaning experiments, the applied pressure fields, both in the standard protocol and the time-reversal enhanced protocol, were characterized by measuring the pressure field point-by-point with a calibrated hydrophone (Brüel & Kjær, 8103) at the same height above the vessel floor as the height of the transducers that were coupled onto the container. The spatial-peak temporal-peak intensity can be interpreted as the focus of the field and the temporal-peak pressure at the target location reflects the cleaning effort. Therefore, by mapping temporal-peak intensities spatially one can determine the acoustic field in both cases and by comparing the measured temporal peak pressures at the target location one can try to predict which protocol should have better cleaning efficiency. Moreover, the acoustic field of the time-reversal enhanced protocol shows ability of TR to focus at the desired location. The measured focus with the standard protocol is at the center of the container, corresponding to the simulation prediction. Similarly, the measured focus with the TR enhanced protocol is at the desired target point, as predicted by theory. The temporal peak-negative-pressures at the target point with standard and time-reversal enhanced protocols were −550 kPa and −1030 kPa, respectively. Therefore, based on this experimental result, the time-reversal enhanced protocol should produce higher cleaning effect.

**Fig. 6:**
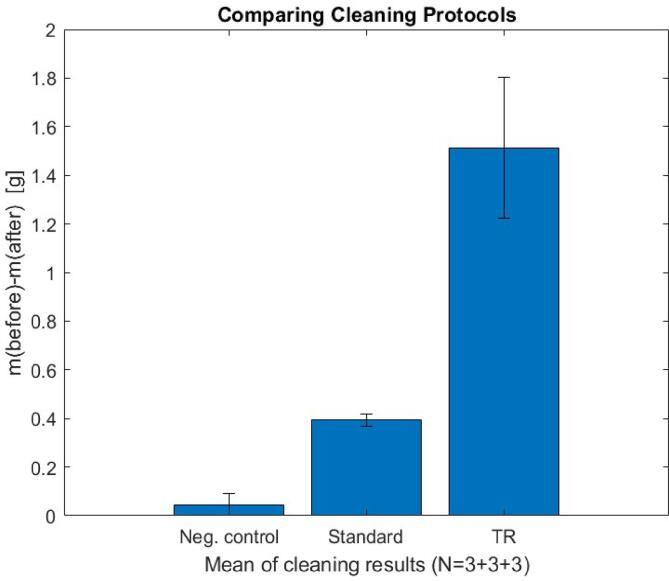
Experimental results of two different cleaning protocols and the negative control. The negative control did not alter the mass of the fouled pipe, indicating that fouling was not significantly removed. The time-reversal enhanced protocol removed more than three times more fouling (1.5 g) compared to the standard protocol (0.4 g), implying the time-reversal enhanced protocol provided higher cleaning efficiency.

To determine the cleaning efficiency, three different protocols were compared to each other by measuring the mass difference of the fouled pipe before and after applying each protocol. In the negative control protocol, the pipe covered by fouling was placed in water at the target location for 60 s. In the standard protocol, the pipe was situated in the same location and sonicated for 60 s with 20-cycle sine wave bursts. In the time-reversal enhanced protocol, the sonication was conducted with FEM-simulated, time-reversed, multitapped signals, having the same 60 s sonication time, burst length, and electric input energy as the standard protocol. Each protocol was repeated for three samples and the means of the mass differences before and after the sonication are shown in Fig. 6. Based on the results, the negative control protocol did not remove fouling significantly, as expected. Comparing the standard protocol to the time-reversal enhanced protocol, the latter removed three times more fouling by mass. Therefore, the TRM protocol provided higher cleaning efficiency than the standard protocol.

## 4 Discussion

The introduced method opens up new possibilities for cleaning complex structures that one cannot access from within, by focusing cleaning power with a time-reversal process. Its application to the current ultrasonic cleaning protocols improves the efficiency of the ultrasonic cleaning. These are the first results from applying a time-reversal focusing technique for ultrasound cleaning. As described in the introduction, there are several parameters in the high-power regime of ultrasonic cleaning, such as bandwidth, number of transducers, and reciprocity in the non-linear regime, whose impact on the time-reversal need more theoretical and experimental investigation. The benefit of using time-reversal instead of other focusing techniques is that this approach does not require exact information about the impedance contrast of the medium if the forward-propagation can be conducted either experimentally or *in silico*. Therefore time-reversal is usually suitable for focusing e.g. sound in complex structures. Conducting forward-propagation experimentally can be challenging if there is no access to the target point. Simulating the forward-propagation can solve this problem, but creating the corresponding simulation model can be time-consuming. Moreover, there is always a mismatch between the simulation model and reality (e.g. the propagation path might change, if the propagation medium is too different in the simulation model), making efficient focusing nontrivial.

The experiments were done using Plexiglas structures, which allowed us to verify our hypothesis and to demonstrate the approach of combining time-reversal and ultrasound cleaning. Still, our laboratory setup differs from industrial cases in terms of container material and size. Since steel is a common material in many industrial systems, and since sound propagation is different in steel and in Plexiglas, validation is still needed in steel structures.

In our case, only one pipe was present in the container during each sonication. As a proof-of-concept experiment, this setup configuration allowed us to compare the TRM method to the standard protocol, but it did not fully mimic common industrial heat exchangers that usually carry several internal pipes.

In the described experiments, we used Ca(OH)_2_ fouling on the pipes, which is a common fouling type [2]. This suffices for our current purpose, since our goal was to show that combining the time-reversal process and multitapping with ultrasound cleaning techniques may permit efficient cleaning of complex structures. It should be possible to improve the cleaning efficiency by increasing the number of transducers or by using longer sonication times and higher acoustic power.

The challenge of using time reversal with high-power ultrasound arises from the narrow bandwidth of the high-power transducers and from non-linear effects present when the transducers are driven at high power levels. In many ultrasound TRM applications, recording and transmitting sound waves require phased, broadband transducer arrays to achieve a tight focus and to have weak grating lobes [23], [24]. Both these features are important in order to provide maximum cleaning power to the desired cleaning location. Unfortunately, common power amplifiers may be inefficient for amplifying broadband signals [25]. In addition, high power can potentially break the reciprocity principle of time reversal through nonlinear phenomena [26], which may prevent re-focusing onto the original source in the back-propagation phase [27]. All these factors can potentially limit the cleaning efficiency in some cases. Still, there is experimental evidence that time-reversal focusing in nonlinear regime can be efficient [28].

## 5 Conclusions

We introduced an enhanced ultrasonic fouling-removal method with spatial focusing ability by using simulated and time-reversed signals. The method showed promise for extending the current ultrasonic cleaning protocols to remove fouling in complex structures. We compared a standard ultrasound cleaning protocol with a time-reversal-based approach. From this comparison we concluded that with the same electric energy and sonication time, more effective cleaning is obtained with the proposed time reversal-enhanced protocol. In our method, time-reversed signals were produced using a FEM-simulation and the sonication was performed with four narrowband transducers. Our results support the hypothesis that the TRM could be used to focus cleaning power, which could enable cleaning complex structures with improved efficiency.

### CRediT authorship contribution statement

**Joonas Mustonen:** Methodology, Validation, Formal analysis, Investigation, Writing – original draft, Visualization. **Oskari Tommiska:** Methodology, Investigation, Visualization, Writing – review & editing. **Axi Holmström:** Methodology, Supervision, Writing – review & editing. **Timo Rauhala:** Software, Conceptualization, Resources, Writing – review & editing. **Petro Moilanen:** Conceptualization, Writing – review & editing. **Maria Gritsevich:** Conceptualization, Writing – review & editing. **Ari Salmi:** Conceptualization, Resources, Supervision, Project administration, Writing – review & editing. **Edward Hæggström:** Conceptualization, Resources, Supervision, Project administration, Writing – review & editing.

## Declaration of Competing Interest

The authors declare that they have no known competing financial interests or personal relationships that could have appeared to influence the work reported in this paper.
